# Similarities and differences of chemical compositions and physical and functional properties of adjuvant system 01 and army liposome formulation with QS21

**DOI:** 10.3389/fimmu.2023.1102524

**Published:** 2023-01-25

**Authors:** Carl R. Alving, Mangala Rao, Gary R. Matyas

**Affiliations:** Laboratory of Adjuvant and Antigen Research, U.S. Military HIV Research Program, Walter Reed Army Institute of Research, Silver Spring, MD, United States

**Keywords:** ALFQ, AS01, monophosphoryl lipid A, QS21 (QS-21) saponin, liposomes, phospholipase A 2, vaccine adjuvant

## Abstract

A vaccine adjuvant known as Adjuvant System 01 (AS01) consists of liposomes containing a mixture of natural congeners of monophosphoryl lipid A (MPL^®^) obtained from bacterial lipopolysaccharide, and a tree saponin known as QS21. Two vaccines containing AS01 as the adjuvant have been licensed, including a malaria vaccine (Mosquirix^®^) approved by World Health. Organization and European Medicines Agency for use in sub-Saharan Africa, and a shingles vaccine (Shingrix^®^) approved by the U.S. Food and Drug Administration. The success of the AS01 vaccine adjuvant has led to the development of another liposomal vaccine adjuvant, referred to as Army Liposome Formulation with QS21 (ALFQ). Like AS01, ALFQ consists of liposomes containing monophosphoryl lipid A (as a synthetic molecule known as 3D-PHAD^®^) and QS21 as adjuvant constituents, and the polar headgroups of the liposomes of AS01 and ALFQ are similar. We compare here AS01 with ALFQ with respect to their similar and different liposomal chemical structures and physical characteristics with a goal of projecting some of the likely mechanisms of safety, side effects, and mechanisms of adjuvanticity. We hypothesize that some of the side effects exhibited in humans after injection of liposome-based vaccines might be caused by free fatty acid and lysophospholipid released by enzymatic attack of liposomal phospholipid by phospholipase A_2_ at the injection site or systemically after injection.

## 1 Introduction

In 1986, scientists at the Walter Reed Army Institute of Research (WRAIR), together with collaborators, reported that liposomes containing lipid A derived from Gram-negative bacterial lipopolysaccharide (LPS) exhibited potent adjuvant activity for inducing antibodies to a human malaria sporozoite antigen; because of this it was proposed that liposomes containing lipid A might be developed as the adjuvant element of a malaria vaccine (1). Nine fundamental strategies and advantages of using liposomes as carriers of antigens and adjuvants for vaccines were summarized later by WRAIR scientists (2, 3).

### 1.1 Adjuvant system 01

Commercial development of a liposomal malaria vaccine, known as RTS,S/AS01 (or Mosquirix^®^), was originally created as part of a collaboration initiated in 1984 between SmithKline Beecham (now known as GlaxoSmithKline, or GSK), and WRAIR (and other collaborators) to develop a vaccine to malaria (4). Inventive activity by GSK subsequently resulted in emergence of a novel liposomal vaccine adjuvant formulation known as AS01, which comprises liposomes containing both several congeners of monophosphoryl lipid A (MPLA) derived from bacterial lipopolysaccharide (MPL^®^) and a saponin fraction (QS21, also known as QS-21) extracted from the bark of the soap bark tree *(Quillaja saponaria)* (5–7). It should be noted that MPLA is used in this article as an abbreviation for “monophosphoryl lipid A”, but as described later, different chemical structures of MPLA compounds are known by trademarked terms, including MPL^®^ (GSK) or 3D-PHAD^®^ (Avanti Polar Lipids). The AS01 formulation is now an integral part of two approved vaccines, a malaria vaccine (Mosquirix^®^) approved by the European Medicines Agency and the World Health Organization, which is directed against *Plasmodium falciparum* malaria in sub-Saharan Africa (8–10), and a shingles vaccine (Shingrix^®^) licensed by the U.S. Food and Drug Administration for commercial use in the U.S., and which is also licensed in other countries. AS01 has also been used as the adjuvant in a phase 2b vaccine trial against tuberculosis (11).

### 1.2 Army liposome formulation with QS21

During the period from 1986 until now, scientists at WRAIR continued to utilize liposomes containing MPLA, which were referred to as Walter Reed liposomes, but are now known as Army Liposome Formulation (ALF), as an adjuvant platform for different types of vaccines, including malaria, HIV-1, and several types of cancer [reviewed in (12)]. However, the emergence of the innovative and successful creation by GSK of AS01 as a commercial adjuvant platform for multiple vaccines led the WRAIR scientists to explore whether liposomes containing MPLA and QS21, but with bulk liposomal lipid compositions that differed from AS01, could lead to the creation of unique adjuvant formulations. It was reasoned that fundamental differences in ALF-type liposomal lipid composition compared to AS01 might enable the use of liposomes containing MPLA and QS21 by WRAIR as unique adjuvants for military vaccine development.

**Figure 1:**
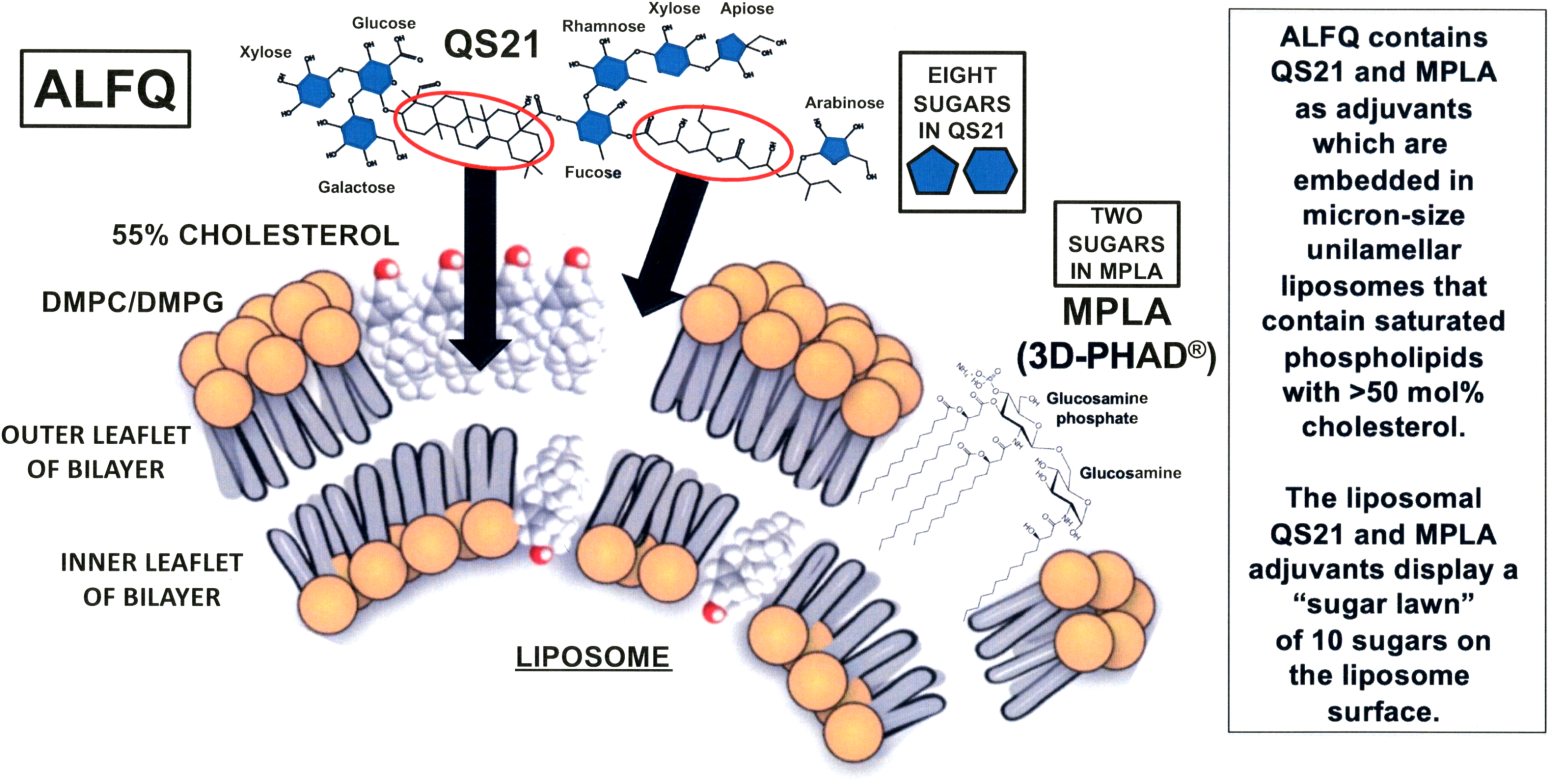
Schematic illustration of the hypothetical process of formation of ALFQ. Liposomes (known as ALF55) containing DMPC, DMPG, 55 mol% cholesterol relative to the phospholipids, and MPLA, are mixed with soluble QS21 to form ALFQ. The triterpenoid region of QS21 approaches the surface of ALF55 (left oval with arrow) and then binds directly to cholesterol where it forms channels, and the flexible hydrocarbon region between fucose and arabinose (right oval with arrow) approaches and then becomes embedded in an unknown manner in the hydrophobic region of ALF55. Due to a subsequent fusion event, large (micron sized) unilamellar vesicles are formed by cannibalization of ALF55 nanoparticles.

In 2015, basic research on liposomal membrane structures revealed that dramatic differences in the visibility of membrane-associated cholesterol and MPLA, and even different sizes of liposomes containing both MPLA and QS21, could be achieved by changes in the composition of liposomal bulk phospholipids and cholesterol (13, 14) (**Figure 1**).

The invention and patenting of Army Liposome Formulation with QS21 (ALFQ) (15) has enabled the testing of numerous different completed, ongoing, or projected human trials utilizing ALFQ as the vaccine adjuvant (16). Seven current ongoing or planned vaccine trials containing ALFQ as an adjuvant include two different vaccines to *Plasmodium falciparum* malaria (17–19), two different HIV-1 vaccines (20, 21), a vaccine to traveler’s diarrhea caused by *Campylobacter jejuni* (22), a vaccine to SARS-CoV-2 which targets multiple variants to prevent COVID-19 (23), and a phase 1 trial which is planned to test a universal influenza vaccine candidate utilizing unconjugated peptides as antigens (24).

## 2 Discussion

### 2.1 Mechanisms of adjuvanticity

Among the many possible mechanisms of adjuvanticity that are common to AS01 and ALFQ, several proposed mechanisms stand out: *first*, binding of one or more of the ten sugars at the polar regions of the MPLA and QS21 molecules to any of numerous glycan-binding lectin receptors, such as C-type lectins, leading to cellular uptake and various types of intracellular activity (25–28); *second*, binding of the liposomal MPLA to toll-like receptor 4 (TLR4), possibly serving as a TLR4 agonist with activation of TLR4 on dendritic cells and other cells (29); *third*, high affinity binding of the triterpene ring of saponins, such as QS21, to cholesterol causes dramatic changes in the nanoarchitecture of liposomes or other particles containing cholesterol, including punching of permeability channels in the cholesterol region and causes creation of unusual shapes that might be viewed as danger (or damage) associated molecular patterns (DAMPs) (13, 14, 30–32); and *fourth*, QS21 and lipid A each is associated with complex intracellular immunomodulatory effects, including induction of inflammasomes and caspases (33, 34).

With regard to the above theoretical mechanisms, it should be noted that although liposomal MPLA is known to have potent adjuvant activity, and exhibits considerable safety in humans (12, 16, 35–38), it might (or might not) serve as a TLR4 agonist as mentioned above. The precise role of the head-group of liposomal MPLA by itself, as opposed to the hydrophobic region of liposomal MPLA, as a TLR4 agonist is still under investigation (39). In addition, it is not clear how the entire liposomal lipid A molecule could interact directly with the TLR4-MD-2 receptor complex on a target cell because liposomal lipid A is firmly and deeply embedded in the liposomes, and interactions with the TLR4-MD-2 complex presumably would require complicated interactions with the entire lipid A molecule independently of the liposomal membrane (39–41). It has been further reported that intracellular bacterial lipopolysaccharide (which includes lipid A as the major active site) binds to an unknown intracellular receptor other than TLR4, leading to activation of noncanonical caspase-11 (42–44). As with binding and TLR4-MD-2 receptor activation by liposomal lipid A, the possible intracellular recognition of liposomal MPLA, which is deeply embedded in the liposomal membrane, by the intracellular MPLA receptor has not been investigated.

With respect to the roles of the sugars in the hydrophilic polar region of QS21, there are seven different individual sugars (galactose, two of xylose, glucose, fucose, rhamnose, apiose, and arabinose), and these are individually located at eight different sites on the polar region of the QS21 portion of the framework of both AS01 and ALFQ; and MPLA contributes two additional polar sugars (in the form of a disaccharide consisting of glucosamine connected through a β(1–6) linkage to glucosamine-4’-phosphate). These ten carbohydrates constitute a sort of sugar lawn at the surfaces of AS01 or ALFQ particles, and the sugars thus might enable the binding and uptake of the liposomeembedded adjuvants and associated antigens by immune cells, such as macrophages, dendritic cells, and other cells that display sugar-specific mammalian lectins on their surfaces that recognize pathogens as foreign objects (45).

### 2.2 Differences between the liposomes and liposomal adjuvants in AS01 and ALFQ

Despite the above similarities, the liposomes in AS01 and in ALFQ have dramatically different chemical compositions and physical properties; these differences involve important areas, namely, liposomal phospholipid composition and fatty acyl saturation, total and relative amounts of cholesterol content, and liposome size (16). The bulk phospholipid of AS01 liposomes consists of a net neutral zwitteronic unsaturated phospholipid, dioleoyl phosphatidylcholine (DOPC); in contrast, ALFQ contains two different phospholipids, both of which contain saturated fatty acyl groups: neutral zwitterionic dimyristoyl phosphatidylcholine (DMPC) and anionic dimyristoyl phosphatidylglycerol (DMPG). The cholesterol (CHOL) differences between AS01 and ALFQ depend in part on the injection volume of AS01 when compared to ALFQ, but the total CHOL content of ALFQ is at least >20-fold higher by weight when compared to AS01, and the mole percent ratios of liposomal CHOL to phospholipid are greatly different, 33.7% for AS01 and 55% for ALFQ (16). With respect to size, the median diameter of AS01 liposomal particles is uniformly approximately 100 nm (46). In contrast, addition of QS21 to nano-sized ALF55 liposomes containing 55% CHOL to form ALFQ results in a remarkable liposomal fusion event, leading to a polydisperse final size distribution of unilamellar liposomes ranging from approximately 50 nm to as large as 30,000 nm (14). The fate of AS01 particles after intramuscular injection in mice has been reported previously (47), and studies on the effects of particle size on tissue distribution of polydisperse ALFQ vs. AS01-like nanoparticles containing 3D-PHAD^®^ instead of MPL^®^ (32) are planned in animal models. Effects, if any, of the size of ALFQ on vaccine safety have not yet been directly determined, but results from the seven ongoing or planned clinical vaccine trials with ALFQ described earlier might provide some guidance in the future.

Another notable difference between AS01 and ALFQ lies in the chemical structures of the MPLA adjuvants. MPL^®^, which is obtained by GSK by extraction of multiple congeners of MPLA from *Salmonella minnesota* R595 lipopolysaccharide, contains hexa-acylated, and penta-acylated MPLA (each of which can serve as a TLR4 agonist based on human monocyte model cell culture studies), and also tetra-acylated MPLAs (which can serve as a TLR4 antagonist in human model cell cultures) and tri-acylated MPLAs (which can serve as a TLR4 antagonist in human model cell culture) (48). In contrast, ALFQ contains 3D-PHAD^®^ (from Avanti Polar Lipids), which is a pure penta-acylated synthetic lipid A (which can serve as a TLR4 agonist in human model cell culture) (47). Although the disaccharide polar headgroups are identical in MPL^®^ and 3D-PHAD^®^, the above authors note that the presence of both TLR4 agonists and antagonists that exist in the hydrophobic regions of the multiple MPLA congeners in MPL^®^ differentiate it from 3D-PHAD^®^ (48). The later paper further suggested, based on human model cell culture studies, that differences in the hydrophobic regions of MPLA might explain differences of immunostimulatory or inflammatory activities of different forms of liposomal MPLA after intramuscular injection as vaccine adjuvants in humans.

From all of this, it is clear that the physical and chemical characteristics of the hydrophobic regions of AS01 and ALFQ are quite different, with regard both to the bulk liposomal lipids and to the chemical structures of the hydrophobic regions of MPLA. Here we propose some ways in which differences in the liposomal phospholipid and cholesterol compositions might influence functional characteristics of AS01 and ALFQ as vaccine adjuvants.

### 2.3 Potential differential susceptibility of AS01 and ALFQ to attack by phospholipase A_2_

There are many different groups and types of phospholipase A_2_ (PLA_2_), including secreted PLA_2_, cytosolic PLA_2_, calcium-independent PLA_2_, lipoprotein-associated PLA_2_, lysosomal PLA_2_, and adipose PLA_2_. Despite the diversity, all types of PLA_2_ enzymatically remove the *sn*-2 fatty acyl chain of the phospholipid, resulting in generation of free fatty acid (FFA) and lysophospholipid (lysoPL) (49, 50). Among the many types of PLA_2_ described in mammalian tissues, some may cause release of saturated as well as unsaturated fatty acids. However, liposomes that contain unsaturated phospholipids (such as in AS01) have less tightly arranged fatty acyl chains than saturated chains (such as in ALFQ) because of kinks in the chains at the double bonds; these discontinuities diminish the ability to form strong van der Waals bonds which weaken exponentially with the distance between the chains (51). Liposomes having greater phospholipid fatty acyl fluidity due to unsaturated fatty acyl chains may have greater susceptibility to enzymatic attack by PLA_2_. However, addition of cholesterol in the liposomal bilayer can increase the stability of the liposomes and might harden the particles against PLA_2_ attack (52). As an example, inclusion of 35 mol% cholesterol in liposomes containing DMPC (the phospholipid in ALFQ), or with other similar saturated phospholipids, completely inhibited enzymatic attack by pancreatic PLA_2_ (53). In addition, liposomes containing cholesterol together with unsaturated plant phospholipids were more susceptible to tumor cell-associated PLA_2_ attack than were liposomes having saturated plant phospholipids, and liposomes containing purified FFA or lysoPL exhibited direct cytotoxic activities against cultured human tumor cells or normal cells (54–57). Lysophosphatidylcholine (LPC) is recognized as a DAMP (58), and interestingly, with macrophages from mice injected with LPS (TLR primed macrophages), but not with non-TLR primed macrophages, it was reported that LPC promoted inflammatory cytokines and cytotoxicity that were dependent on the release of extracellular ATP (another DAMP) from the cells (59).

**Figure 2:**
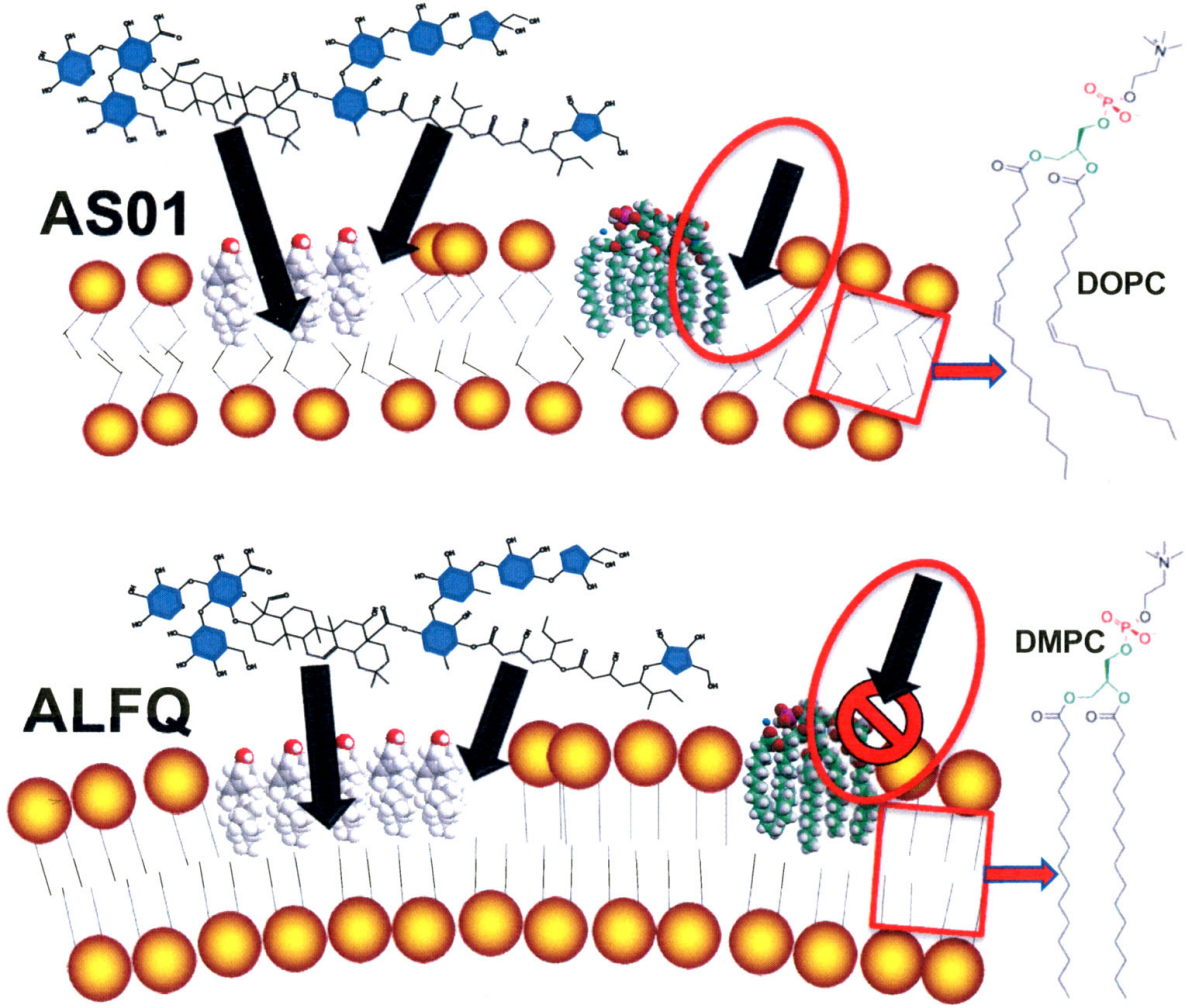
Theoretical comparative vulnerabilities of AS01 and ALFQ to toxic effects of MPLA and to attack by PLA_2_. Unsaturated phospholipid fatty acids disrupt van der Waals interactions and cause greater fluidity of hydrophobic region of liposomes containing DOPC than those containing DMPC and DMPG. The endotoxic activity of MPLA resides exclusively in the hydrophobic region of the MPLA (16). Increased fluidity of DOPC liposomes in AS01 (box in AS01 image) could provide access to the toxic hydrophobic region of MPLA and to attack by PLA_2_ (oval with arrow in AS01 image). Tight binding of saturated phospholipid fatty acids (DMPC and DMPG) caused by strong van der Waals interactions in ALFQ (box in ALFQ image) might prevent exposure of the hydrophobic region of MPLA (oval with arrow in ALFQ image).

From a practical standpoint, one theoretical effect of using liposomes containing unsaturated phospholipids, such as in AS01, when compared to liposomes containing saturated DMPC phospholipids, such as in ALFQ, might be that AS01 is more susceptible to attack and degradation by certain types of PLA_2_ than ALFQ (**Figure 2**). Injection of liposomes containing a substrate for attack by PLA_2_ might result in local cytotoxic effects due to local accumulation of FFA and lysoPL, and it seems likely that this could lead to local inflammatory reactions at the site of injections. Although, the actual existence and degree of such reactions, if they occur, can only be observed directly after human injection, but mitigation, if needed, could be considered with inhibitors of PLA_2_ (60).

### 2.4 Effects of AS01 and ALFQ as constituents of human vaccines

Although AS01 has been utilized as an adjuvant constituent in numerous human clinical trials, it is most noted for achieving regulatory approval as a constituent in a shingles vaccine (Shingrix^®^) in adults, and a *P. falciparum* malaria vaccine (Mosquirix^®^) in infants. As previously mentioned, ALFQ is being tested as an adjuvant in seven phase 1 trials, but there are no direct studies underway to compare the potency of AS01 vs. ALFQ as an adjuvant constituent in a vaccine containing the same antigen. As noted earlier, although AS01 and ALFQ both comprised liposomes containing QS21 and different types of MPLA compounds as adjuvants, it has been suggested that a mixture of natural congeners serving either as TLR4 agonists or antagonists in MPL^®^, as opposed to synthetic PHAD^®^ serving only as a TLR4 agonist, might reflect some differences of adjuvanticity and safety aspects of AS01 when compared to ALFQ (48). However, in view of the previously mentioned differences in the chemistry and structure of the bulk phospholipids of liposomes between AS01 and ALFQ, it seems reasonable also to speculate on the possibility of certain safety and reactogenicity aspects related to liposomal bilayer membrane fluidity and nanoarchitecture that might be observed in humans.

### 2.5 Predicted side effects in humans after intramuscular injection of vaccines containing AS01 or ALFQ

As most of us know, intramuscular injection can sometimes be uncomfortable. Indeed, in one double-blinded study, among 1312 individuals aged 50-59 who were injected intramuscularly with a placebo consisting of 0.5 ml of saline, 14% experienced pain, 15% myalgia, 20% fatigue, 22% headache, 7% shivering, 3% fever, and 11% gastrointestinal symptoms within 1-7 days of the injection (61). However, certain individuals who are injected with a vaccine containing a recombinant protein antigen and AS01 as an adjuvant instead of saline alone, sometimes experience more discomfort. In the above double-blinded study, a separate parallel group of 1315 individuals among those in the same age range who were injected with a vaccine to shingles containing a recombinant protein and AS01 adjuvant (Shingrix^®^, GSK), 88% experienced pain, 57% myalgia, 57% fatigue, 51% headache, 36% shivering, 28% fever, and 24% gastrointestinal symptoms (61). After a phase 1 trial with another antigen (HBsAg) adjuvanted with AS01, increased reactogenicity also occurred when compared to placebo injection, especially after two vaccine injections, and it was concluded that “Adjuvants like AS01B increase the immunogenicity of vaccines and generally cause increased transient reactogenicity compared with Alum.” (62). In the latter study, it was further speculated that “…similar innate immune signals may underlie adjuvant reactogenicity and immunogenicity.” It occurs to us that it seems unlikely that strongly increased reactogenicity is necessarily required in order for a vaccine to display strongly increased potency. For example, in a phase 1 vaccine trial comparing seven different adjuvants, each vaccine utilizing a recombinant gp120 envelope antigen, alum-adsorbed ALF-type liposomes containing MPLA evoked no greater local or systemic toxicity than alum alone (37), but induced much stronger immune responses to the gp120 antigen than with alum alone (38).

In the first-in-human trial utilizing ALFQ as an adjuvant (FMP013/ALFQ) in ten adult subjects, the antigen consisted of a soluble circumsporozoite recombinant malaria protein that was adjuvanted with ALFQ (17, 18). Two different vaccine doses were used: five subjects were given a low injection dose of 0.5 ml having 20 µg of antigen and ALFQ containing 100 µg of MPLA and 50 µg of QS21; and five subjects were given a higher injection dose of 1.0 ml with 40 µg of antigen, 200 µg of MPLA, and 100 µg of QS21. The low dose side effects were quite mild: the most severe effect was moderate redness at injection site and headache in one individual at one visit. The high dose was slightly more reactogenic, but all observed events were graded mild or moderate and there were no severe adverse events. The RTS,S recombinant protein antigen in the Mosquirix^®^ (GSK) malaria vaccine also contains circumsporozoite repeat sequence epitopes that were present in the FMP013/ALFQ vaccine, and it was further concluded in the FMP013/ALFQ study: “Both groups [low and high dose] exhibited robust humoral and cellular immunological responses, and compared favorably with historical responses reported for RTS,S/AS01” (17). In other words, the FMP013/ALFQ vaccine might have immunogenicity against malaria that is similar or greater than that exhibited by RTS,S/AS01 malaria vaccine in clinical studies in adults.

It should be noted that when AS01 is used for the shingles vaccine (Shingrix^®^), a single 0.5 ml dose contains 50 µg of MPLA and 50 µg of QS21. However, because of the low degree of reactogenicity of ALFQ found in the FMP013/ALFQ study, higher levels of MPLA (200 µg) and QS21 (100 µg) are currently being employed for certain other vaccines, and we anticipate that this higher dose of adjuvant might enable higher immunogenicity of the vaccine without causing increased side effects.

In summary, based on the above physical and chemical similarities and differences between the liposomes, we conclude that AS01 and ALFQ probably have similar adjuvant potency. However, we also hypothesize that ALFQ liposomes are more rigid due to stronger van der Waals forces between the fatty acyl chains in the hydrophobic region of the lipid bilayer, and are thus less permeable than AS01 liposomes. With ALFQ this might restrict access to the phospholipid fatty acyl chains by PLA_2_, leading to decreased release of toxic FFA and lysoPL; and it also might result in less visibility of fatty acyl chains that might promote toxic inflammatory activity in ALFQ. Overall, we predict that the initial low reactogenicity observed with the FMP013/ALFQ vaccine will also be observed with other future vaccines containing ALFQ.

## Data availability statement

The original contributions presented in the study are included in the article/supplementary material. Further inquiries can be directed to the corresponding author.

## Author contributions

All authors listed have made a substantial, direct, and intellectual contribution to the work and approved it for publication.

## References

[1] Alving CR Richards RL Moss J Alving LI Clements JD Shiba T. Effectiveness of liposomes as potential carriers of vaccines: Applications to cholera toxin and human malaria sporozoite antigen. Vaccine (1986) 4(3):166–72. doi: 10.1016/0264-410x(86)90005-8 3532603

[2] Alving CR. Liposomes as carriers for vaccines. In: Ostro M, editor. Liposomes: From biophysics to therapeutics. New York: Marcel Dekker (1987). p. 195–218.

[3] Alving CR Richards RL. Liposomes containing lipid a: A potent nontoxic adjuvant for a human malaria sporozoite vaccine. Immunol Lett (1990) 25(1-3):275–9. doi: 10.1016/0165-2478(90)90127-c 2283158

[4] Laurens MB. RTS,S/AS01 vaccine (Mosquirix™): An overview. Hum Vaccin Immunother (2020) 16(3):480–9. doi: 10.1080/21645515.2019.1669415 PMC7227679 31545128

[5] Garcon NM Friede M. Vaccines containing a saponin and a sterol. international application number: PCT/EP96/01464. World Intellectual Property Organization International Publication Number WO 96/33739 (1996). Available at: https://patentscope.wipo.int/search/en/detail.jsf?docId=WO1996033739.

[6] Vandepapeliere P. Vaccine compositions comprising a saponin adjuvant. United States patent 10143745 (2018). GlaxoSmithKline Biologicals, S.ARixensart (BE).

[7] Vandepapeliere P. Vaccine compositions comprising a saponin adjuvant. United States patent 10039823 (2018). GlaxoSmithKline Biologicals, S.ARixensart (BE).

[8] EMA. Mosquirix: Opinion on medicine for use outside EU (2015). Available at: https://www.ema.europa.eu/en/opinion-medicine-use-outside-EU/human/mosquirixopiniondetails-section (Accessed September 19, 2022).

[9] GSK. Fact sheet: RTS,S malaria vaccine candidate (Mosquirix™) (2016). Available at: https://www.malariavaccine.org/files/content/page/files/RTSS%20vaccine%20candidate%20Factsheet_FINAL.pdf (Accessed September 19, 2022).

[10] GSK. WHO grants prequalification to GSK’s mosquirix – the first and only approved malaria vaccine (2022). Available at: https://www.gsk.com/en-gb/media/press-releases/who-grants-prequalificationto-gsk-s-mosquirix-the-first-and-only-approved-malaria-vaccine/ (Accessed September 19, 2022).

[11] Tait DR Hatherill M van der Meeren O Ginsberg AM Van Brakel E Salaun B. Final analysis of a trial of M72/AS01E vaccine to prevent tuberculosis. N Engl J Med (2019) 381(25):2429–39. doi: 10.1056/NEJMoa1909953 31661198

[12] Alving CR Rao M Steers NJ Matyas GR Mayorov AV. Liposomes containing lipid A: An effective, safe, generic adjuvant system for synthetic vaccines. Expert Rev Vaccines (2012) 11(6):733–44. doi: 10.1586/erv.12.35 22873129

[13] Beck Z Matyas GR Alving CR. Detection of liposomal cholesterol and monophosphoryl lipid a by QS-21 saponin and limulus polyphemus amebocyte lysate. Biochim Biophys Acta (2015) 1848(3):775–80. doi: 10.1016/j.bbamem.2014.12.005 25511587

[14] Beck Z Matyas GR Jalah R Rao M Polonis VR Alving CR. Differential immune responses to HIV-1 envelope protein induced by liposomal adjuvant formulations containing monophosphoryl lipid a with or without QS21. Vaccine (2015) 33(42):5578–87. doi: 10.1016/j.vaccine.2015.09.001 26372857

[15] Alving CR Beck Z. inventors; The Government of the United States as Represented by the Secretary of the Army, assignee. Non-toxic Adjuvant Formulation Comprising a Monophosphoryl Lipid A (MPLA)-Containing Liposome Composition and a Saponin, United States patent 10434167 (2019).

[16] Alving CR Peachman KK Matyas GR Rao M Beck Z. Army liposome formulation (ALF) family of vaccine adjuvants. Expert Rev Vaccines (2020) 19(3):279–92. doi: 10.1080/14760584.2020.1745636 PMC7412170 32228108

[17] Hutter JN Robben PM Lee C Hamer M Moon JE Merino K. First-in-human assessment of safety and immunogenicity of low and high doses of plasmodium falciparum malaria protein 013 (FMP013) administered intramuscularly with ALFQ adjuvant in healthy malaria-naïve adults. Vaccine (2022) 40(40):5781–90. doi: 10.1016/j.vaccine.2022.08.048 36055874

[18] ClinicalTrials.gov. A trial for the study of falciparum malaria protein 013 administered via intramuscular injection, in: Healthy adults (2020). Available at: https://clinicaltrials.gov/ct2/show/NCT04268420 (Accessed October 8, 2022).

[19] ClinicalTrials.gov. Phase 1 clinical trial with controlled human malaria infection (CHMI) for safety, protective efficacy, and immunogenicity of plasmodium falciparum malaria protein (FMP014) administered intramuscularly with ALFQ healthy malaria-naïve adults (2020). Available at: https://clinicaltrials.gov/ct2/show/NCT04296279?term=alfq&draw=2&rank=3 (Accessed October 8, 2022).

[20] ClinicalTrials.gov. HIV Vaccine in HIV-uninfected adults (2020). Available at: https://clinicaltrials.gov/ct2/show/NCT04658667?term=alfq&draw=2&rank=6 (Accessed October 8, 2022).

[21] ClinicalTrials.gov. A phase 1 randomized study to evaluate the safety, tolerability, and immunogenicity of ranging doses of ALFQ adjuvant in a candidate HIV vaccine containing A244 and B.65321 (2022). Available at: https://clinicaltrials.gov/ct2/show/NCT05423418?term=alfq&cond=hiv-1&draw=2&rank=1 (Accessed October 8, 2022).

[22] ClinicalTrials.gov. First-in-Human safety and immunogenicity evaluation of an intramuscular campylobacter jejuni conjugate vaccine (CJCV2) with and without army liposome formulation containing QS-21 (ALFQ) (2022). Available at: https://clinicaltrials.gov/ct2/show/NCT05500417?term=alfq&cond=Campylobacter+Jejuni+Infection&draw=2&rank=1 (Accessed October 8, 2022).

[23] ClinicalTrials.gov. A PHASE 1, randomized, double-blind, placebo-controlled study to evaluate the safety, tolerability, and immunogenicity of ranging doses of SARS-COV-2-Spike-Ferritin-Nanoparticle (SPFN_1B-06-PL) vaccine with army liposomal formulation QS21 (ALFQ) for prevention of COVID-19 in healthy adults (2021). Available at: https://clinicaltrials.gov/ct2/show/NCT04784767 (Accessed October 8, 2022).

[24] Sei CJ Rao M Schuman RF Daum LT Matyas GR Rikhi N. Conserved influenza hemagglutinin, neuraminidase and matrix peptides adjuvanted with ALFQ induce broadly neutralizing antibodies. Vaccines (Basel) (2021) 9(7):698. doi: 10.3390/vaccines9070698 34202178 PMC8310080

[25] Marciani DJ. New Th2 adjuvants for preventive and active immunotherapy of neurodegenerative proteinopathies. Drug Discovery Today (2014) 19(7):912–20. doi: 10.1016/j.drudis.2014.02.015 24607730

[26] Marciani DJ. Is fucose the answer to the immunomodulatory paradox of quillaja saponins? Int Immunopharmacol (2015) 29(2):908–13. doi: 10.1016/j.intimp.2015.10.028 26603552

[27] Marciani DJ. Elucidating the mechanisms of action of saponin-derived adjuvants. Trends Pharmacol Sci (2018) 39(6):573–85. doi: 10.1016/j.tips.2018.03.005 29655658

[28] Lacaille-Dubois MA. Updated insights into the mechanism of action and clinical profile of the immunoadjuvant QS-21: A review. Phytomedicine (2019) 60:152905. doi: 10.1016/j.phymed.2019.152905 31182297 PMC7127804

[29] Casella CR Mitchell TC. Putting endotoxin to work for us: Monophosphoryl lipid a as a safe and effective vaccine adjuvant. Cell Mol Life Sci (2008) 65(20):3231–40. doi: 10.1007/s00018-008-8228-6 PMC2647720 18668203

[30] Bangham AD Horne RW Glauert AM Dingle JT Lucy JA. Action of saponin on biological cell membranes. Nature (1962) 196:952–5. doi: 10.1038/196952a0 13966357

[31] Paepenmüller T Müller-Goymann CC. Influence of quil a on liposomal membranes. Int J Pharm (2014) 475(1-2):138–46. doi: 10.1016/j.ijpharm.2014.08.007 25107288

[32] Singh P Beck Z Matyas GR Alving CR. Saturated phospholipids are required for nano- to micron-size transformation of cholesterol-containing liposomes upon QS21 addition. J Liposome Res (2019) 29(3):247–50. doi: 10.1080/08982104.2018.1538239 30350748

[33] Lamkanfi M Dixit VM. Mechanisms and functions of inflammasomes. Cell (2014) 157(5):1013–22. doi: 10.1016/j.cell.2014.04.007 24855941

[34] Marty-Roix R Vladimer GI Pouliot K Weng D Buglione-Corbett R West K. Identification of QS-21 as an inflammasome-activating molecular component of saponin adjuvants. J Biol Chem (2016) 291(3):1123–36. doi: 10.1074/jbc.M115.683011 PMC4714196 26555265

[35] Fries LF Gordon DM Richards RL Egan JE Hollingdale MR Gross M. Liposomal malaria vaccine in humans: a safe and potent adjuvant strategy. Proc Natl Acad Sci U.S.A. (1992) 89(1):358–62. doi: 10.1073/pnas.89.1.358 PMC48236 1729706

[36] Heppner DG Gordon DM Gross M Wellde B Leitner W Krzych U. Safety, immunogenicity, and efficacy of plasmodium falciparum repeatless circumsporozoite protein vaccine encapsulated in liposomes. J Infect Dis (1996) 174(2):361–6. doi: 10.1093/infdis/174.2.361 8699067

[37] McElrath MJ. Selection of potent immunological adjuvants for vaccine construction. Semin Cancer Biol (1995) 6(6):375–85. doi: 10.1016/1044-579x(95)90007-1 8938276

[38] Rao M Onkar S Peachman KK White Y Trinh HV Jobe O. Liposome-encapsulated human immunodeficiency virus-1 gp120 induces potent V1V2-specific antibodies in humans. J Infect Dis (2018) 218(10):1541–50. doi: 10.1093/infdis/jiy348 29893872

[39] Cochet F Peri F. The role of carbohydrates in the lipopolysaccharide (LPS)/Toll-like receptor 4 (TLR4) signalling. Int J Mol Sci (2017) 18(11):2318. doi: 10.3390/ijms18112318 29099761 PMC5713287

[40] Poltorak A Ricciardi-Castagnoli P Citterio S Beutler B. Physical contact between lipopolysaccharide and toll-like receptor 4 revealed by genetic complementation. Proc Natl Acad Sci U S A. (2000) 97(5):2163–7. doi: 10.1073/pnas.040565397 PMC15771 10681462

[41] Maeshima N Fernandez RC. Recognition of lipid A variants by the TLR4-MD-2 receptor complex. Front Cell Infect Microbiol (2013) 3:3. doi: 10.3389/fcimb.2013.00003 23408095 PMC3569842

[42] Hagar JA Powell DA Aachoui Y Ernst RK Miao EA. Cytoplasmic LPS activates caspase-11:implications in TLR4-independent endotoxic shock. Science (2013) 341(6151):1250–3. doi: 10.1126/science.1240988 PMC3931427 24031018

[43] Kayagaki N Wong MT Stowe IB Ramani SR Gonzalez LC Akashi-Takamura S. Noncanonical inflammasome activation by intracellular LPS independent of TLR4. Science (2013) 341(6151):1246–9. doi: 10.1126/science.1240248 23887873

[44] Yang J Zhao Y Shao F. Non-canonical activation of inflammatory caspases by cytosolic LPS in innate immunity. Curr Opin Immunol (2015) 32:78–83. doi: 10.1016/j.coi.2015.01.007 25621708

[45] Manabe N Yamaguchi Y. 3D structural view of pathogen recognition by mammalian lectin receptors. Front Mol Biosci (2021) 8:670780. doi: 10.3389/fmolb.2021.670780 34113651 PMC8185196

[46] Jin J Tarrant RD Bolam EJ Angell-Manning P Soegaard M Pattinson DJ. Production, quality control, stability, and potency of cGMP-produced plasmodium falciparum RH5.1 protein vaccine expressed in drosophila S2 cells. NPJ Vaccines (2018) 3:32. doi: 10.1038/s41541-018-0071-7 30131879 PMC6098134

[47] Didierlaurent AM Collignon C Bourguignon P Wouters S Fierens K Fochesato M. Enhancement of adaptive immunity by the human vaccine adjuvant AS01 depends on activated dendritic cells. J Immunol (2014) 193(4):1920–30. doi: 10.4049/jimmunol.1400948 25024381

[48] Wang YQ Bazin-Lee H Evans JT Casella CR Mitchell TC. MPL adjuvant contains competitive antagonists of human TLR4. Front Immunol (2020) 11:577823. doi: 10.3389/fimmu.2020.577823 33178204 PMC7596181

[49] Vasquez AM Mouchlis VD Dennis EA. Review of four major distinct types of human phospholipase A_2_ . Adv Biol Regul (2018) 67:212–8. doi: 10.1016/j.jbior.2017.10.009 PMC5807221 29248300

[50] Dennis EA. Allosteric regulation by membranes and hydrophobic subsites in phospholipase A2 enzymes determine their substrate specificity. J Biol Chem (2022) 298(5):101873. doi: 10.1016/j.jbc.2022.101873 35358512 PMC9079178

[51] Seu KJ Cambrea LR Everly RM Hovis JS. Influence of lipid chemistry on membrane fluidity:tail and headgroup interactions. Biophys J (2006) 91(10):3727–35. doi: 10.1529/biophysj.106.084590 PMC1630490 16950848

[52] Nakhaei P Margiana R Bokov DO Abdelbasset WK Jadidi Kouhbanani MA Varma RS. Liposomes: Structure, biomedical applications, and stability parameters with emphasis on cholesterol. Front Bioeng Biotechnol (2021) 9:705886. doi: 10.3389/fbioe.2021.705886 34568298 PMC8459376

[53] Op den Kamp JA Kauerz MT van Deenen LL. Action of pancreatic phospholipase A2 on phosphatidylcholine bilayers in different physical states. Biochim Biophys Acta (1975) 406(2):169–77. doi: 10.1016/0005-2736(75)90001-2 1191645

[54] Jett M Chudzik J Alving CR Stanacev NZ. Metabolic fate of liposomal phosphatidylinositol in murine tumor cells: implications for the mechanism of tumor cell cytotoxicity. Cancer Res (1985) 45(10):4810–5. 4027970

[55] Jett M Alving CR. Tumoricidal effects of liposomes containing phosphatidylinositol or phosphatidylcholine. Methods Enzymol (1987) 141:459–66. doi: 10.1016/00766879(87)41092-6 3298971

[56] Kigoshi S Ito R. High levels of free fatty acids in lymphoid cells, with special reference to their cytotoxicity. Experientia (1973) 29(11):1408–10. doi: 10.1007/BF01922845 4761256

[57] Jung S Lee S Lee H Yoon J Lee EK. Oleic acid-embedded nanoliposome as a selective tumoricidal agent. Colloids Surf B Biointerfaces (2016) 146:585–9. doi: 10.1016/j.colsurfb.2016.06.058 27424089

[58] Shao Y Nanayakkara G Cheng J Cueto R Yang WY Park JY. Lysophospholipids and their receptors serve as conditional DAMPs and DAMP receptors in tissue oxidative and inflammatory injury. Antioxid Redox Signal (2018) 28(10):973–86. doi: 10.1089/ars.2017.7069 PMC5849278 28325059

[59] Ismaeel S Qadri A. ATP release drives inflammation with lysophosphatidylcholine. Immunohorizons (2021) 5(4):219–33. doi: 10.4049/immunohorizons.2100023 33911018

[60] Batsika CS Gerogiannopoulou AD Mantzourani C Vasilakaki S Kokotos G. The design and discovery of phospholipase A2 inhibitors for the treatment of inflammatory diseases. Expert Opin Drug Discovery (2021) 16(11):1287–305. doi: 10.1080/17460441.2021.1942835 34143707

[61] GSK. FDA Package insert for shingrix (2021). Available at: https://www.fda.gov/media/108597/download (Accessed October 11, 2022).

[62] Burny W Marchant A Hervé C Callegaro A Caubet M Fissette L. Inflammatory parameters associated with systemic reactogenicity following vaccination with adjuvanted hepatitis b vaccines in humans. Vaccine (2019) 37(14):2004–15. doi: 10.1016/j.vaccine.2019.02.015 30850240

